# A physiotherapy protocol* for stroke patients in acute hospital settings: expert consensus from the Brazilian early stroke rehabilitation task force

**DOI:** 10.1055/s-0045-1806924

**Published:** 2025-04-22

**Authors:** Iara Maso, Gustavo José Luvizutto, Jéssica Mariana de Aquino Miranda, Carla Ferreira do Nascimento, Luana Aparecida Miranda Bonome, Elen Beatriz Pinto, Fabiane Maria Klitzke, Ricardo Machado Souza, Carla Heloisa Cabral Moro, Rodrigo Bazan, Pedro Antonio Pereira de Jesus, Eduardo de Melo Carvalho Rocha, Cesar Minelli, Sheila Ouriques Martins, Jussara Almeida de Oliveira Baggio

**Affiliations:** 1 Hospital Geral Roberto Santos, Unidade de AVC, Salvador BA, Brazil.; 2 Escola Bahiana de Medicina e Saúde Pública, Grupo de Pesquisa Comportamento Motor e Reabilitação Neurofuncional, Salvador BA, Brazil.; 3 Universidade Federal do Triângulo Mineiro, Departamento de Fisioterapia Aplicada, Uberaba MG, Brazil.; 4 Universidade Estadual Paulista, Faculdade de Medicina de Botucatu, Botucatu SP, Brazil.; 5 Hospital Municipal São José, Programa de Residência Multiprofissional em Neurologia, Joinville SC, Brazil.; 6 Universidade de São Paulo, Faculdade de Medicina de Ribeirão Preto, Hospital das Clínicas, Unidade de AVC, Ribeirão Preto SP, Brazil.; 7 Hospital São José, Unidade de AVC, Joinville SC, Brazil.; 8 Universidade Federal da Bahia, Instituto de Ciências da Saúde, Salvador BA, Brazil.; 9 Santa Casa de São Paulo, Faculdade de Ciências Médicas, São Paulo SP, Brazil.; 10 Hospital Carlos Fernando Malzoni, Matão SP, Brazil.; 11 Universidade de São Paulo, Faculdade de Medicina de Ribeirão Preto, Programa de Pós-Graduação do Departamento de Neurociências e Ciências do Comportamento, Ribeirão Preto SP, Brazil.; 12 Hospital de Clínicas de Porto Alegre, Porto Alegre RS, Brazil.; 13 Universidade Federal do Rio Grande do Sul, Porto Alegre RS, Brazil.; 14 Rede Brasil AVC, Porto Alegre RS, Brazil.; 15 World Stroke Organization, Geneva, Switzerland.; 16 Universidade Federal de Alagoas, Curso de Medicina, Arapiraca AL, Brazil.

**Keywords:** Stroke, Stroke Rehabilitation, Physical Therapy Modalities, Early Ambulation

## Abstract

The present protocol provides general recommendations based on the best evidence currently available for physiotherapists to use as a guide for the care of stroke patients during hospitalization. The Brazilian Early Stroke Rehabilitation Task Force, comprising physical therapy experts and researchers from different Brazilian states, was organized to develop this care protocol based on a bibliographical survey, including meta-analyses, systematic reviews, clinical trials, and other more recent and relevant scientific publications. Professionals working in stroke units were also included in the task force to ensure the practicality of the protocol in different contexts. This protocol provides guidance on assessment strategies, safety criteria for the mobilization of patients with stroke, recommendations for mobilization and proper positioning, as well as evidence-based practices for treatment during hospitalization, including preventive measures for shoulder pain and shoulder-hand syndrome. The protocol also provides information on the organization of the physiotherapy service at stroke units, guidelines for hospital discharge, and quality indicators for physiotherapy services. We have included detailed activities that can be performed during mobilization in the supplementary material, such as postural control training, sensory and perceptual stimulation, task-oriented training, and activities involving an enriched environment. The protocol was written in a user-friendly format to facilitate its application in different social and cultural contexts, utilizing resources readily available in most clinical settings.

## INTRODUCTION

In 2022, stroke was the leading cause of death in Brazil and has remained among the main global causes of hospitalization and disability in recent years. 1 2 Among neurological disorders, it is considered to represent the greatest rehabilitation demand for the global population. 3 Recent data has shown that 612,646 individuals aged 50 and over were hospitalized for stroke in Brazil between January 2020 and November 2022, while hospital morbidity was almost 5% for this age group during the same period. 2

Given its epidemiological importance and the disparities found in the type of care offered, there have been growing efforts aimed at stroke prevention, increasing survival rates, and reducing disabilities caused by stroke. 4 5 These actions include an increase in access to proper care in both the hyperacute (first 24 hours) and acute phases (up to 7 days), in addition to evidence and guidelines that directly assist the population affected by this disease. 5 6 7 8

Although there is consensus regarding the importance of physical therapy after stroke, some aspects have not yet been fully established. Recent studies have pointed out that intensive early mobilization, if started within the first 24 hours after stroke, does not contribute to a favorable functional outcome; however, gaps remain regarding the ideal frequency and intensity of motor training during acute stroke rehabilitation. 9

National and international rehabilitation guidelines for patients with stroke represent a major advance in scientific knowledge and care. 7 10 11 12 13 These guidelines address the timing of mobilization but do not discuss the ideal dose (frequency, duration, and intensity) or the safety criteria for mobilization. Certain characteristics of the acute and hyperacute stroke phases, such as clinical and hemodynamic instability, bleeding risk, and care for cerebral hypoperfusion, 14 15 as well as other aspects that permeate hospitalization, pose specific challenges for physical therapists providing assistance to this population.

In Brazil, specialization courses in neurofunctional physiotherapy are generally aimed at the rehabilitation of patients with neurological diseases, without a specific focus on patients with stroke, and several do not address all particularities of the hyperacute and acute phases. Additionally, a significant number of physiotherapists working in hospitals have specializations in respiratory physiotherapy and intensive care, without specific training in neurorehabilitation. In view of the heterogeneity found in professional training and hospital care, there is a need to develop an evidence-based protocol to guide the physical therapeutic approach for hospitalized stroke patients within the context of the Brazilian social and public health realities. This protocol may also direct the implementation of training programs specifically aimed at physiotherapists working in units that treat patients in the acute and subacute phases after stroke.

A working group of physical therapy experts and researchers from different Brazilian states was organized to develop a care protocol based on a bibliographical survey, including meta-analyses, systematic reviews, clinical trials, and other more recent and relevant scientific publications. In addition to evidence found in the literature, the working group also considered the experience of professionals working in stroke units, aiming to render the protocol viable for administration in Brazil.

This protocol aims to optimize physical therapeutic assistance for patients with stroke during hospitalization. It was developed specifically for Stroke Units but can also be used as a guideline to care for stroke patients hospitalized in other units, such as emergency wards and intensive care units (ICUs). It is worth emphasizing that services catering to stroke patients should adopt the stroke unit model, given the robust evidence of improved outcomes in both the short and long term. 16 The protocol can be used for individuals who are hospitalized after an ischemic or hemorrhagic stroke, whether undergoing reperfusion therapy or not (intravenous chemical thrombolysis/mechanical thrombectomy).

## PROTOCOL DEVELOPMENT METHODOLOGY

Experts in stroke rehabilitation with clinical experience in the field were invited to form the Brazilian Early Stroke Rehabilitation Task Force. Brazil is a continental country with different socioeconomic realities; for this reason, the group was composed by professionals from different geographical regions and various specialties.

The first meeting of the task force took place during the Global Stroke Alliance, in August 2022, with the attendance of stroke rehabilitation experts, representatives from the Ministry of Health of Brazil, and people with lived experience with stroke. During the meeting, a thematic panel was held where the main gaps related to the rehabilitation of stroke patients were identified, and it was decided to start by developing national rehabilitation protocols.

Following the identification of task force priorities, a group responsible for the preparation of this protocol was formed. It consisted of 8 physiotherapists chosen based on the following criteria: living in different regions of Brazil, having publication in the field, and/or having at least 5 years of clinical experience in stroke units.

The group conducted a literature search until December 2022. The search details for this scoping review are presented in **Supplementary Material I** (available at https://www.arquivosdeneuropsiquiatria.org/wp-content/uploads/2025/02/ANP-2024.0096-Supplementary-Material-1.pdf ). Subsequently, the group discussed the evidence in the field and determined the main objective and subtopics of the protocol. Between January and June 2023, the writing of the protocol took place, involving monthly online meetings for discussions. This co-production was based on shared decision-making, mutual respect, and learning. At the onset of the protocol development, guidelines were established and shared as a reference document, outlining the Brazilian Early Stroke Rehabilitation Task Force's objectives, expectations, and communication methods. Building on this foundation, the decision-making process adhered to criteria previously defined within the group, allowing all members the opportunity to provide input. Following these criteria, the coordinator identified key points requiring group discussion, and each member had the chance to express their position, drawing on both scientific evidence and professional experience. Discrepancies were then resolved through voting, with consensus achieved by majority vote. In all topics, consensus was reached, and no disagreements persisted after the voting process. The preestablished relationships within the group were crucial to the successful completion of the project.

Once the protocol writing was finalized, we invited a panel of reviewers, which included an expert physiotherapist in stroke rehabilitation, stroke neurologists, and a physiatrist. After the review process, we made necessary changes, and the final manuscript underwent further revision by representatives of the Brazilian Association of Neurofunctional Physiotherapy and the Scientific Department of Neurological Rehabilitation of the Brazilian Academy of Neurology. The physiotherapists who developed the protocol approved the final version of the manuscript.

**Figure 1:**
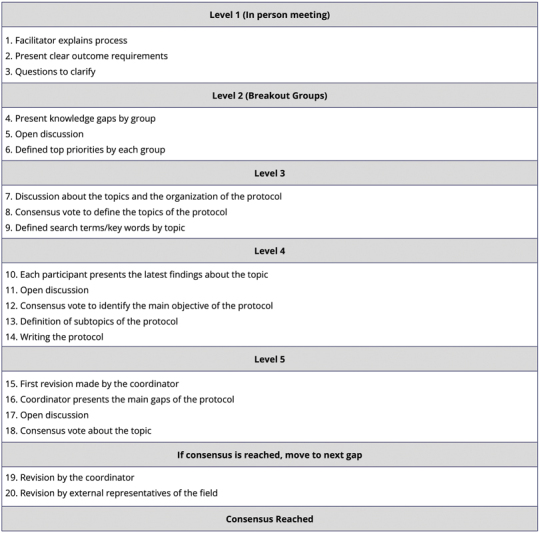
Structured decision-making process used for in-person and virtual consensus meetings.

Figure 1 presents the structured decision-making process used for the in person and virtual consensus meetings. Additionally, **Supplementary Figure I** (online only) shows the profile of physiotherapists involved in the development of the protocol, along with the profile of the panel of reviewers ( **Supplementary Material I** ; online only).

## ASSESSMENT OF STROKE PATIENTS IN ACUTE HOSPITAL SETTINGS

Before the intervention, patients must be carefully evaluated to adequately plan physical therapy procedures, which will be performed according to the individual's functional level.

Physical therapeutic evaluation may follow the evaluation form templates for each service, with the recommendation of including the information described in **Supplementary Table 1** (online only) ( **Supplementary Material I** ; online only).

The inclusion of validated clinical scales with medium- and long-term prognostic values is recommended during hospitalization, allowing for quantitative assessment of the patient's evolution during physical therapy. The suggested tools were selected through a consensus of a group of experts, based on the scientific literature. 7 17 18 The criteria for classifying the instruments as *Highly Recommended Assessment Tools* were: evidence-based support of their use in acute or subacute phase of stroke patients, validation in Brazilian Portuguese, adequate measurement properties, and ease of application in clinical practice. 19 20 21 22 23 24 The instruments categorized as *Recommended Assessment Tools* followed the same criteria, but require more time to administer. Additionally, we included among the *Recommended Assessment Tools* some tests that are quick and easy to apply but have limitations preventing their use with all poststroke patients. For example, aphasic patients may be unable to complete the Borg Rating of Perceived Exertion 25 and Star Cancelation Test. 26

Table 1^27^,^28^ shows selected tools that were classified as *Highly Recommended Assessment Tools* , while Table 2 29 shows tools that were classified as *Recommended Assessment Tools* . Paid assessment tools were not included, as this would hinder their implementation in Brazil given the country's socioeconomic context.

**Table 1:** Highly recommended stroke rehabilitation assessment tools

Assessment tool	Purpose	ICF domain	Description	Specialized training	When to apply
National Institutes of Health Stroke Scale (NIHSS) 17 19	Measures the severity of neurological symptoms	Body Function	11 items assessing level of consciousness, conjugate gaze, visual fields, facial palsy, motor strength upper limbs, motor strength lower limbs, ataxia, sensory, language, dysarthria, extinction or inattention. Scores range from 0 to 42 points, with higher scores indicating greater severity of the stroke. Link to training: https://www.youtube.com/watch?v=pbUOytrTQ8I	Required	Daily
Modified Rankin Scale (mRS) 19 27	Categorizes level of functional independence	Activity	mRS is a disability scale that includes gait, basic activities and usual activities assessment. The mRS score ranges from 0 to 6, with 0 - Asymptomatic and 6–Death. Link to training: https://www.youtube.com/watch?v=pbUOytrTQ8I	Required	At admission, collect previous mRS At hospital discharge
Hospital Mobility Scale (HMS) 20 21	Evaluates the mobility of stroke patients in the hospital environment	Activity	The HMS evaluate three mobility tasks: sitting, standing and gait. This scale is based on the amount of assistance in performing these mobility tasks (performs independently, needs help from 1 person, needs help from 2 people, or fails to perform the task). The total score ranges from 0 to 12, and the higher the score, the greater the degree of dependence. Link to access the scale free of charge: http://www5.bahiana.edu.br/index.php/fisioterapia/article/view/3199	Not required	Daily
10 Meter Walk Test 17 28	Measures walking speed	Activity	The time to cover the given distance is recorded.	Not required	When the patient starts walking At hospital discharge

Abbreviation: ICF, International Classification of Functioning, Disability and Health.

**Table 2:** Recommended stroke rehabilitation assessment tools

Assessment tool	Purpose	ICF domain	Description	Specialized training	When to apply
Barthel Index (BI) 19	Evaluates autonomy in activities of daily living (ADLs)	Activity	There are 10 items that assess activities related to clothing, nutrition, personal hygiene, and transfers. Each item is scored as 0, 5, 10, or 15, resulting in a total score of 100. A higher score indicates greater functional independence.	Not required	In the first evaluation At hospital discharge
Fugl-Meyer 29	Evaluates the motor function of the upper and lower limbs	Body Function	The subscale assesses motor recovery of the upper and lower limbs. The maximum score for the upper limb is 66, and for the lower limb is 34. The higher the score, the better the motor function. Link to training: https://www.gu.se/en/neuroscience-physiology/fugl-meyer-assessment	Required	In the first evaluation At hospital discharge
Postural Assessment Scale for Stroke (PASS) 29	Evaluates poststroke postural control	Activity	There are 12 items divided into postural maintenance (5 items) and postural changes (7 items). Each item is scored from 0 to 3, with a maximum score of 36 points. The higher the score, the better the postural function.	Not required	In the first evaluation At hospital discharge
Borg Rating of Perceived Exertion 25	Measure aerobic capacity	Body Function	A scale from 6 to 20 is used for individuals to assess their perception of the intensity of the prescribed exercise. Aphasic patients may be unable to complete this test.	Not required	Daily
Star Cancelation Test 26	Evaluates spatial neglect	Body Function	Screening test for spatial neglect. The test consists of 52 large stars, 13 small stars, words, and letters among the stars. The instruction is to mark all the small stars. The sheet is positioned along the patient's midline. Aphasic patients may be unable to complete this test.	Not required	In the first evaluation At hospital discharge
Timed Up and Go 29	Evaluates mobility	Activity	The patient starts sitting on a chair, upon the evaluator's command, walks 3 m, returns, and sits back on the chair.	Not required	When the patient starts walking At hospital discharge

Abbreviation: ICF, International Classification of Functioning, Disability and Health.

In services with a reduced number of physical therapists, we suggest that at least the following scales be applied:

- National Institutes of Health Stroke Scale (NIHSS): score daily.
- Hospital Mobility Scale (HMS): score daily.
- Modified Rankin Scale (mRS): score at hospital discharge.

The selection of the NIHSS, mRS, and HMS was based on recommendations from the scientific literature regarding the use of these scales in clinical practice 17 19 20 21 27 . Additionally, we considered that all of them have been validated for the Brazilian population, with adequate measurement properties, and are easy to apply in clinical settings.

We recommend using the mRS, as this tool is quick to administer, free of charge, widely accepted in clinical practice, and commonly used in international clinical trials involving stroke patients. Due to its widespread use, we recommend applying the mRS both prior to the stroke (data already collected for reperfusion therapy decisions 30 ) and at hospital discharge. The objective of the mRS is to assess overall functionality; however, its interpretation in a hospital setting is limited. 17 Therefore, services with greater availability of professionals can replace the mRS with the Barthel Index (BI), another widely used scale for assessing stroke patients, 31 particularly in evaluating the *activity* component of the International Classification of Functioning (ICF). Since it requires more time to administer, we classified the BI as a recommended scale. If possible, we suggest that these daily life activity assessment scales be administered by a multidisciplinary team, dividing the workload and enhancing team integration. The scales are easy to administer, so the main barrier would be the availability of professionals' time.

The Recommended Assessment Tools, described in Table 2 , can be used with patients who require a more detailed evaluation of specific aspects. For example, a patient with specific upper-limb demands can be assessed with the Fugl-Meyer 29 if the team has sufficient time available. Similarly, patients with particular demands related to postural control can be evaluated with the Postural Assessment Scale for Stroke (PASS). 29 The Borg Rating of Perceived Exertion 25 can be used with patients with respiratory issues, while the Star Cancellation Test 26 can be applied to those with perceptual deficits. However, as we discussed previously, these last two tests may not be appropriate for aphasic patients. Therefore, we do not recommend applying all scales to assess every patient; rather, we advocate for the individual assessment of patients and the tailored selection of instruments based on their specific needs.

## DESCRIPTION OF THE PROCEDURES

The procedures described in this protocol are:

- Mobilization.
- Positioning in bed.
- Preventive measures for shoulder pain and shoulder-hand syndrome.

NOTE: The description of respiratory procedures is not part of the objectives of this protocol and must follow local protocols for respiratory physical therapy (oxygen therapy, tracheostomy, and invasive and noninvasive mechanical ventilation protocols). If the service lacks these protocols, you may refer to the Brazilian and International Guidelines for the same. 32 33 34 35 36 37 38 39 40 41

Neurocritical patients who require admission to an intensive care unit (ICU) have peculiarities that are not described in this protocol because they do not represent the profiles of patients in stroke units. We suggest reading papers that discuss the mobilization of this specific group of patients. 42 43

At the end of the protocol, we include the following information:

- Organization of the physiotherapy service at stroke units.
- Guidelines for hospital discharge.
- Quality indicators for physiotherapy after a stroke ( **Supplementary Material I** ; online only).
- Postural control training ( **Supplementary Material I** ; online only).
- Sensory and perceptual aspects ( **Supplementary Material I** ; online only).
- Task-oriented training ( **Supplementary Material I** ; online only).
- Enriched environment ( **Supplementary Material I** ; online only).

### Mobilization

#### *Definition of mobilization*

Mobilization was defined by Langhorne et al. as situations in which: “The patient is assisted and encouraged in functional tasks, including activities such as sitting over the edge of the bed, standing up, sitting out of bed and walking.” 44 Therefore, passive exercises performed with patients lying in bed should not be considered as mobilization. During mobilization, it is important for patients to be actively engaged.

#### *Safety criteria for mobilization*

**Figure 2:**
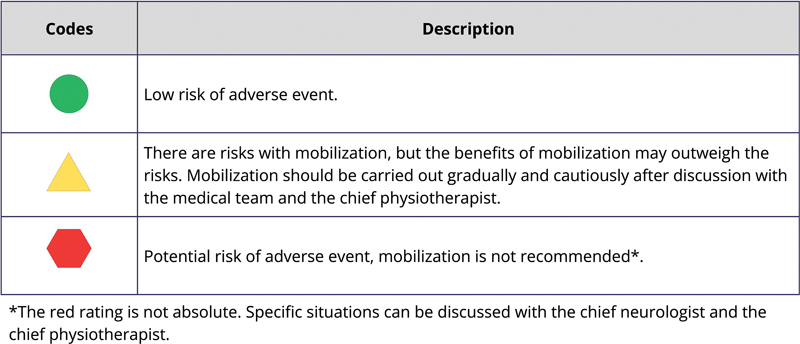
Safety criteria codes.

**Figure 3:**
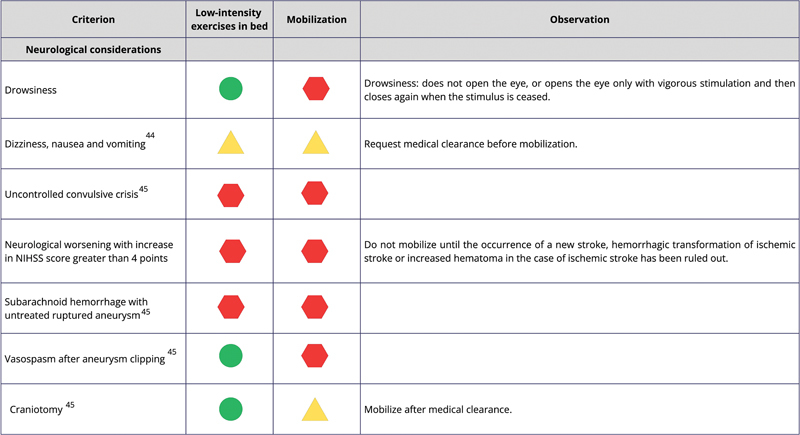
Abbreviation: NIHSS, National Institutes of Health Stroke Scale. Neurological safety considerations.

Figure 2 shows the safety classification codes and descriptions, while Figures 3 4 5 list the criteria to be met by the patients, along with their respective codes, for mobilization and/or low-intensity exercises in bed.

**Figure 4:**
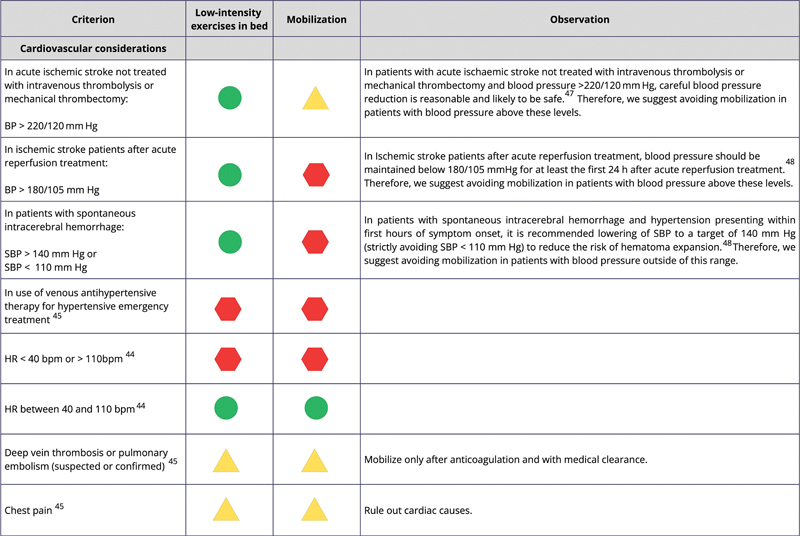
Abbreviations: BP, blood pressure; mmHg, millimeters of mercury; SBP, systolic blood pressure; HR, heart rate; bpm, beats per minute. Cardiovascular safety considerations.

**Figure 5:**
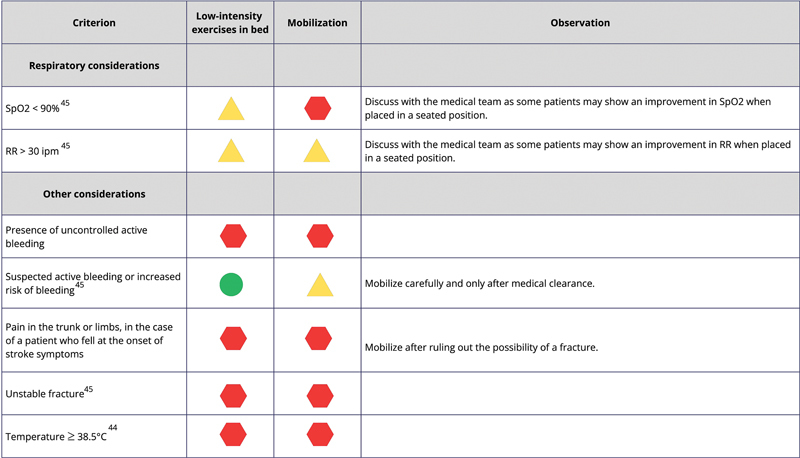
Abbreviations: SpO2, peripheral capillary oxygen saturation; RR, respiratory rate; °C, degrees Celsius. Respiratory and other safety considerations.

For situations in which poststroke population studies were not found, we used the safety criteria to mobilize critically ill patients admitted to the ICU. 45 46

The low-intensity bed exercises mentioned in Figures 3 4 5 were scored between 6 and 10 on the Borg Rating of Perceived Exertion (20-point scale) (RPE 20 ). 25 If the patient is unable to respond to the Borg scale, the physical therapist can observe the patient's signs. Low-intensity exercises are those the patient can perform without difficulty or fatigue, being able to speak during the exercises, and exhibiting minimal or no changes in respiratory rate (RR) or heart rate (HR).

The described safety criteria must always be followed before a patient is mobilized, regardless of whether they are undergoing reperfusion therapy (intravenous chemical thrombolysis/mechanical thrombectomy). For less common clinical situations in stroke units, check the full study by Hodgson et al.. 45 We used the European Stroke Organization guidelines and a systematic review of global stroke guidelines from the World Stroke Organization to define the safety criteria related to blood pressure. 47 48

Most of the safety criteria presented rely on basic assessments and vital signs, which are standard in hospital settings. In situations in which continuous monitoring with individual monitors is unavailable in the unit, we suggest using a portable sphygmomanometer and finger oximeter, along with manually monitoring respiratory and heart rates. Portable devices that use either auscultatory or oscillometric methods of measurement provide reliable blood pressure values. 49 Pulse oximeters, mainly in the middle finger, 50 can be similarly effective in preserving sensitivity to clinically relevant hypoxia. 51 If these devices are also unavailable, clinical and neurological signs such as decreased responsiveness, dizziness, vertigo, nausea, vomiting, headache, pallor, and sweating should be observed. We recommend analyzing each situation on a case-by-case basis and discussing them with the multidisciplinary team.

#### *When to start mobilization*

The recommendations for mobilization in this protocol are primarily based on the results of the A Very Early Rehabilitation Trial (AVERT), which is the largest rehabilitation clinical trial conducted with stroke patients. 52 This multicenter, randomized controlled trial was conducted in 56 stroke units and included 2,104 subjects. 52 The results, published in 2015, demonstrated that early and intensive mobilization within the initial 24 hours reduced the odds of favorable functional outcomes (no or minimal disability according to mRS). Furthermore, the number of serious adverse events or deaths at 3 months poststroke did not differ significantly between the control and intervention groups. 52 Therefore, in 2016, the American Heart Association/American Stroke Association guidelines do not recommend high doses of very early mobilization within the first 24 hours of stroke. 10 Similarly, the United Kingdom Guidelines recommend that mobilization within 24 hours of stroke onset should only be considered for patients who require minimal or no assistance to mobilize. 13

More recently, systematic reviews and meta-analyses published in 2018 and 2020, and including 9 and 6 studies, respectively, have shown findings similar to those of AVERT. 9 53 Consequently, the Australian and New Zealand Clinical Guidelines for Stroke Management strongly advise against initiating intensive out-of-bed activities within 24 hours of stroke onset. 12

The systematic review published in 2018 showed that very early mobilization may reduce the length of hospital stay by about one day. 53 However, the authors emphasize that this result is based on low-quality evidence; therefore, not sufficient to guide practices.

In light of the aforementioned findings, we suggest that patients undergo mobilization between 24 and 48 hours after stroke onset. Patients who score between 0 and 7 on the NIHSS and require minimal or no assistance in walking may be allowed to walk to the bathroom within the first 24 hours. These criteria should be applied to both patients undergoing reperfusion therapy (intravenous chemical thrombolysis/mechanical thrombectomy) and those who are not. Whenever possible, the initial mobilization should be performed by physiotherapists.

The decision of when to begin mobilization should always take into account the safety criteria outlined in Figures 4 5 6 , especially within the first 24 to 48 hours. The presence of dizziness and nausea is categorized in Figure 3 as a relative contraindication for mobilization. However, within the first 24 to 48 hours, it is generally safer to avoid mobilizing these patients, as these symptoms may signal early neurological deterioration. Overall, during the initial two days, it is advisable not to mobilize patients who exhibit conditions marked in yellow (relative contraindications) in the safety criteria.

**Figure 6:**
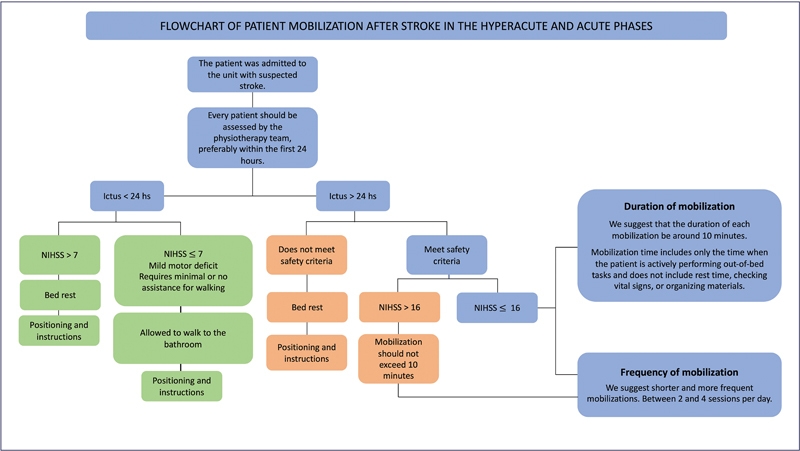
Abbreviation: NIHSS, National Institutes of Health Stroke Scale. Flowchart of patient mobilization after stroke in the hyperacute and acute phases.

The subgroup analysis of the AVERT study did not show any difference between patients who did and those who did not undergo chemical reperfusion therapy. 52 Therefore, the recommendations regarding when to initiate mobilization apply to both groups of patients. However, when feasible, we suggest that patients undergoing chemical and/or mechanical reperfusion therapy be mobilized after undergoing follow-up computed tomography scans and receiving medical clearance, owing to the risk of hemorrhagic transformation. Another important consideration for this group of patients is the need for increased attention to any signs of hemorrhagic transformation, such as headache, dizziness, nausea, vomiting, or worsening in the NIHSS score. If the patient presents with any of the symptoms mentioned above, they should not be mobilized until hemorrhagic transformation is ruled out.

Additionally, patients undergoing mechanical thrombectomy or femoral artery angiography should only be mobilized 6 hours after the procedure or 6 hours after the removal of the sheath or compressive dressing, whichever occurs last. Before mobilizing the patient, the physiotherapist should observe for any bleeding or discomfort at the procedural site. 54 It is worth highlighting that whether the patient has undergone thrombectomy or not does not influence the frequency or duration of mobilization. These factors are determined by the severity of the stroke, as will be discussed in the following sections.

In cases in which patients undergoing reperfusion therapy (intravenous chemical thrombolysis) require airway suctioning, the risk/benefit should be evaluated due to the risk of bleeding.

#### *Frequency of mobilization*

The dose–response analysis from the AVERT study, published in 2016, demonstrated that shorter and more frequent mobilization sessions lead to improved functional outcomes following a stroke. The findings indicated that while keeping the frequency constant, increasing the duration of out-of-bed activities reduced the likelihood of achieving minimal or no disability three months poststroke. These benefits were also evident in patients with more severe strokes (NIHSS > 13.5), in whom a greater number of sessions correlated with more favorable outcomes compared with fewer sessions. 55

Therefore, we suggest that the physiotherapy team distributes the mobilization of each patient at various times of the day (between two and four sessions) with shorter durations. In services with a reduced number of professionals, we recommend that the team aims to achieve at least the goal of two mobilizations per day. These recommendations apply to both mild and severe patients, with either ischemic or hemorrhagic strokes.

Other professional categories, such as nurses, speech therapists, and occupational therapists, can also contribute to increasing the frequency of patient mobilization during their interventions. For instance, patients can be encouraged to sit on the bed, a chair, or an armchair during swallowing evaluations or while being assisted with feeding by speech-language pathologists. Nursing professionals can, whenever possible, motivate patients to walk or use wheelchairs to access the bathroom instead of performing bed baths.

For patients with higher levels of dependency, mobilization may require the assistance of two individuals. In such cases, it is essential for physical therapists to plan their sessions early in the shift and coordinate with other professionals to determine the best time to assist the patient. Additionally, students and family members can contribute to mobilization efforts in units with limited staff availability. This approach aims to achieve the highest possible level of mobility and the ideal frequency of mobilization for each hospitalized patient.

We also recommend that physical therapists perform activities focused on postural control training and task-oriented therapy during mobilization sessions.

#### *Duration of mobilization*

There is no consensus in the literature regarding the duration of mobilization. As previously mentioned, the dose–response analysis for the AVERT study suggests that a higher frequency of mobilizations is preferable to fewer sessions with long duration. 55 In the absence of a study that specifies the ideal duration of mobilization, we estimated this time based on the results of the AVERT study published in 2015, in which the median in the group that showed a better functional outcome was 10 minutes (0.0–18.0) per day spent in out-of-bed activity. 52 Therefore, we suggest that the duration of each mobilization should be ∼ 10 minutes. The session duration does not need to be exactly 10 minutes; it can vary slightly based on the patient's tolerance. The physical therapist should use clinical judgment to assess whether the patient is tolerating the session well or showing signs of fatigue or discomfort. Patients with mild motor deficits may tolerate longer sessions, while those with more significant deficits may need shorter sessions.

A systematic review published in 2020 reported findings consistent with those of AVERT, indicating that early and intensive mobilization may be more harmful for patients with severe and hemorrhagic stroke. 9 Thus, in patients with an NIHSS score greater than 16, we suggest that mobilization should not exceed 10 minutes, and exercise intensity should be low. 56 We also recommend greater attention and care for patients with hemorrhagic stroke and those aged > 80 years. 55 Additionally, mobilization should be approached with extra caution in the first three days poststroke due to greater clinical instability. An ongoing clinical trial, the AVERT DOSE, will elucidate the optimal treatment doses for patients with stroke in the acute phase. 57

Mobilization time includes only the time when the patient is actively performing out-of-bed tasks and does not include rest time, checking vital signs, or organizing materials. There is no time limit for performing personal care tasks and nursing care activities such as going to the bathroom, taking a shower, and sitting down to eat.

Figure 6 presents a flowchart for our proposed physiotherapy management in the acute and hyperacute phases of stroke, including the recommended duration of mobilization according to stroke severity.

#### *When to stop mobilization*

Mobilization should be interrupted, and the patient should be repositioned in bed when: 44

- the physiotherapist or another member of the team determines that mobilization is not tolerated (such as decreased responsiveness, dizziness, vertigo, nausea, vomiting, headache, pallor, sweating, or other reasons);
- heart rate remains > 120 bpm;
- SpO _2_ remains < 90%;
- the patient complains of chest pain (evaluate cardiac causes).

#### *Mobilization plan*

A mobilization plan should be defined according to an individual's functional mobility level, considering the HMS score. Physiotherapists should reassess the level of functional mobility daily to progress mobilization. The main objective of each session should be to achieve a higher level of mobility than in the previous session. It is important to emphasize that mobilization should be performed with the best possible biomechanical alignment to prevent compensation and development of inappropriate motor patterns. 58

During the first three days, low-intensity exercises should be performed with gradual progression based on the patient's tolerance. Table 3 provides a summary of the mobilization plan. In this table, we suggest simpler and less intensive exercises for patients with severe conditions and greater mobility restrictions. As the patient demonstrates functional improvement, the difficulty and intensity of the exercises can be progressively increased. The physiotherapist should use their clinical judgment and closely monitor the patient's tolerance to the exercises, taking into account the previously described safety criteria.

**Table 3:** Mobilization plan for hospitalized patients after stroke

Mobility level	HMS score	Summary of the mobilization plan
1 – Remains only in decubitus	Score 6 in sitting task of the Hospital Mobility Scale	–Exercises in bed
		–Transfer training in bed
2 – Sits with assistance but cannot stand	Score 2 or 4 on the sitting task of the Hospital Mobility Scale. Score 3 on both the standing task and the gait task.	–Transfer training
		–Trunk control training in sitting position
		–Task–oriented training in sitting position
3 – Stands with assistance but cannot walk	Score 1 or 2 on the standing task of the Hospital Mobility Scale. Score 3 on the gait task.	–Transfer training
		–Postural control training in sitting and standing position
		–Task–oriented training in sitting and standing position
4 – Walks with assistance or supervision	Score 1 or 2 on the gait task of the Hospital Mobility Scale.	–Transfer training
		–Postural control training in sitting and standing position
		–Task–oriented training in sitting and standing position
		–Gait training
5 – Walking independently	Score 0 on the gait task of the Hospital Mobility Scale.	–Postural control training in standing position
		–Gait training on uneven terrain, outdoors, maneuvering around or overcoming obstacles

We have included detailed activities that can be performed during mobilization in the **Supplementary Material I** (online only) ( **Appendix 1** . Postural control training; **Appendix 2** . Sensory and perceptual aspects; **Appendix 3** . Task-oriented training; **Appendix 4** . Enriched environment).

Early mobilization is multidisciplinary and requires the collaboration of several health professionals, especially in patients with severe neurological deficits. Cormican et al. identified barriers and facilitators perceived by healthcare professionals in implementing clinical practice guidelines for stroke rehabilitation. 59 Among the most frequently mentioned challenges were organizational factors, including time constraints and limited resources.

To address these issues, we propose strategies to facilitate the application of this protocol in hospitals with limited resources. The mobility training that we suggest can be performed without any equipment, relying solely on the physiotherapist's hands. In hospitals with material resources, instruments such as platforms, balls, cones, and obstacles can be incorporated into physiotherapy sessions to diversify exercises. In more resource-constrained settings, postural control training can be adapted to occur without equipment, utilizing strategies such as narrowing the base of support, closing the eyes, and incorporating directional changes. Additionally, upper-limb activity training can be performed using personal care items available at the patient's bedside, such as moisturizers, toothbrushes, and deodorants. These adaptations make the protocol more accessible and practical across different contexts.

Regarding time constraints, we propose a mobilization session duration of ∼ 10 minutes, which is not overly lengthy for a physiotherapy session. A significant challenge for services with a reduced number of professionals can be achieving the goal of two mobilizations per day. However, this can be facilitated through teamwork, including mobilizations conducted during care provided by other healthcare professionals, such as nursing, occupational therapy, and speech therapy.

### Bed positioning

**Figure 7:**
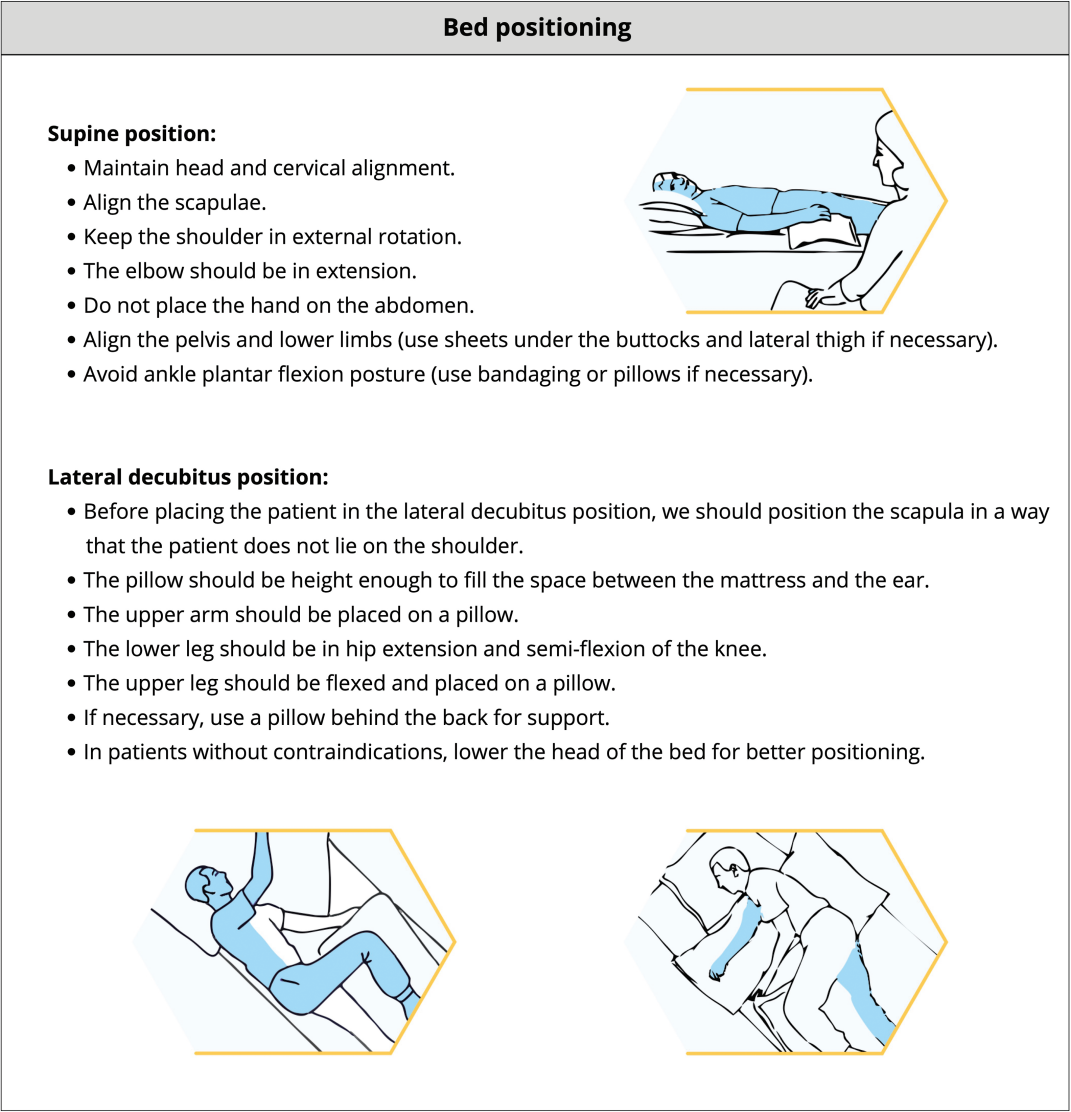
Note: Drawings reprinted from the manual *Shoulder Pain Syndrome after Stroke* , by Associação Brasil AVC, 60 with permission from the authors. Bed positioning.

Therapeutic positioning in a bed, chair, or wheelchair aims to reduce skin damage, limb edema, pain or discomfort, and maximize function while maintaining soft-tissue length. 13 Figure 7 60 presents some considerations related to bed positioning.

Studies using transcranial doppler have shown a clear increase in cerebral blood flow when patients with ischemic stroke are positioned in the lying-flat head position. However, these studies did not assess whether the increased cerebral blood flow improves functional outcomes. 61 The HeadPost trial, a clinical study involving 11,093 patients (85% ischemic stroke and 15% hemorrhagic stroke), compared patients positioned in the lying-flat position versus those in a sitting-up position with the head elevated to at least 30 degrees during the first 24 hours poststroke. 62 The results showed no differences between the groups regarding functional outcomes, mortality, or adverse events such as pneumonia. Patients positioned in the lying-flat position were less likely to maintain this position for 24 hours, potentially due to discomfort caused by the posture. It is worth noting that the HeadPost trial excluded patients with clinical contraindications to lying flat position and that most participants had mild neurological deficits.

A systematic review revealed conflicting results regarding the influence of head positioning on oxygen saturation levels in poststroke patients. 61 Some studies reported higher oxygen saturation levels in the upright head positions compared with the supine position, while others found no changes. The HeadPost trial results demonstrated no differences in oxygen saturation levels between the two groups. 62

Observational studies have shown a reduction in intracranial pressure when the head is elevated in patients with brain injuries. 61 These findings have been used as a rationale for recommending head elevation in patients with acute intracerebral hemorrhage. However, the HeadPost trial, which included 931 patients with hemorrhagic stroke, found no differences in outcomes between patients in the lying-flat position and those in the upright head positions. 62

In light of the evidence from the HeadPost study, 62 clinicians may choose the most comfortable position for patients with ischemic or hemorrhagic stroke in the acute phase, as no differences were observed between the groups. We suggest that patients with mild neurological deficits be positioned in the lying-flat position when feasible, aiming to optimize biomechanical alignment, particularly in the lateral decubitus position. For more severe patients with clinical contraindications to lying flat (e.g., use of nasoenteric feeding tubes, high risk of aspiration, invasive or non-invasive mechanical ventilation, respiratory discomfort, or SpO2 desaturation), who represent a different profile from those included in the HeadPost trial, we recommend maintaining the sitting-up position with the head elevated to at least 30 degrees.

### Prevention of shoulder pain and shoulder-hand syndrome

**Figure 8:**
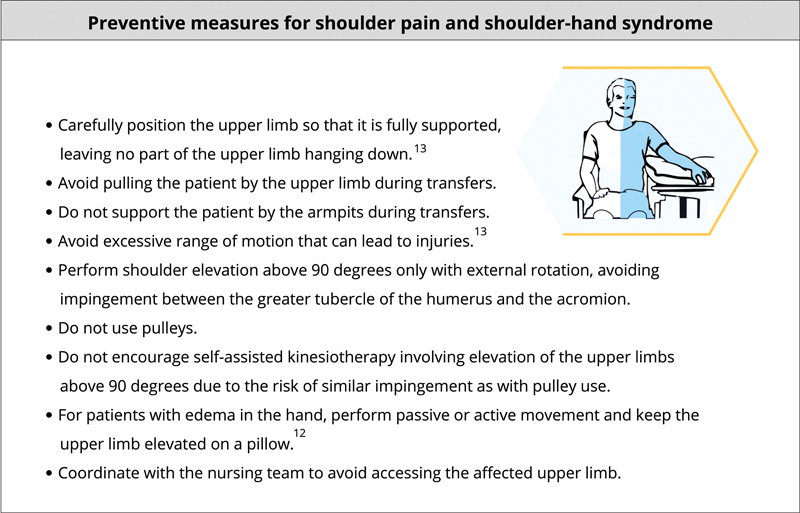
Note: Drawings reprinted from the manual *Shoulder Pain Syndrome after Stroke* , by Associação Brasil AVC, 60 with permission from the authors. Preventive measures for shoulder pain and shoulder-hand syndrome.

Poststroke patients frequently experience complications in the upper limbs, such as shoulder pain and complex regional pain syndrome type I, also known as shoulder-hand syndrome. The prevalence of shoulder pain within 6 months of stroke is estimated to be 17 to 25%. 13 We recommend care strategies for the upper limbs during mobilization to prevent the occurrence of these painful conditions ( Figure 8 ).

Pain levels should be assessed daily, and patient management should involve a multidisciplinary approach. Active motor training is essential to improving function in patients with shoulder pain. 12 This condition can be managed through gentle alignment movements and mobilization with external rotation and abduction. 7 Additionally, handling and positioning recommendations, as described in Figure 8 , are important for pain control when the condition is already established. 12

The physiotherapists should collaborate with the medical team to discuss the need for pharmacological measures for pain management. Patients with severe hypertonicity in hemiplegic shoulder muscles may benefit from Botulinum toxin injections for pain control. 10 National and international guidelines provide further details on the pharmacological options for management of shoulder pain in patients with stroke. 7 10 12 These guidelines also address treatments such as electrostimulation and magnetic stimulation, which are not included in this protocol due to the limited availability of such equipment in most hospitals in Brazil.

## ORGANIZATION OF THE PHYSIOTHERAPY SERVICE AT STROKE UNIT IN BRAZIL

Ordinance Nos. 665/2012 and 800/2015, issued by the Ministry of Health of Brazil, established qualification criteria for hospital establishments as Emergency Care Centers for Patients with Stroke under the Unified Health System (SUS). 63 64 These ordinances classify centers as type I, II (Acute Urgent Care Centers), or III (Comprehensive Stroke Care Units). They describe the physical structure of each type of center and establish a minimum requirement for the number of physiotherapists in stroke centers. In acute centers, a physiotherapist must be present daily without specifying the number of hours; and in comprehensive centers, there should be at least one physiotherapist for every 10 beds available for 6 hours a day. 63 64 However, this number is insufficient to achieve the recommended mobilization frequency of at least two mobilizations per day, as suggested in this protocol. A dose–response analysis of the AVERT study published in 2016 demonstrated that an increased frequency of mobilization leads to better functional outcomes after hospital discharge. 55 The Brazilian Federal Council of Physical Therapy and Occupational Therapy (Conselho Federal de Fisioterapia e Terapia Ocupacional – COFFITO, in Portuguese) Resolution No. 444 of 26/04/2014 for specialized hospital units recommends a minimum of one physiotherapist should be allocated for every 8 to 10 patients for a 6-hour period. 65 The resolution highlights that the specific number of patients to be attended to by each physiotherapist is determined by the chief physiotherapist, considering the level of complexity of the unit and adherence to the principles of dignity and professional ethics.

### Recommendation of this protocol:

To ensure that the patient receives at least 2 physiotherapy sessions per day (1 in the morning and another in the afternoon), a unit with 8 to 10 patients should have at least 1 physiotherapist for a 12-hour period.

In units where physical therapy coverage does not meet the 12-hour standard, we propose some measures to increase the frequency of mobilization:

- To train nursing staff, patients, and caregivers in simple mobilization activities that can be performed between supervised sessions.
- To develop educational programs such as internships and physical therapy residency programs to increase the number of individuals involved in mobilization.
- To assess the redistribution of professionals across different units to meet the demand in areas with a high concentration of stroke cases.
- To prioritize patients with a higher potential for functional recovery or a greater risk of complications due to immobility.
- To monitor quality indicators to support the justification for hiring additional professionals.

Studies investigating the practical application of guidelines in the rehabilitation of patients after a stroke identified that insufficient knowledge and skills among healthcare professionals are significant barriers to the implementation of these guidelines. 59 The authors highlighted that facilitating factors included organizational support, which encompasses training and the presence of local protocols. We believe that the current protocol, which considered the specificities of the Brazilian healthcare system, can assist in guiding clinical practice and facilitate the implementation of a mobilization plan. The protocol can also be used as training material for teams, as it provides easier language and a more practical approach than rehabilitation guidelines.

The physical therapy team should be trained to provide care for stroke patients based on the best available evidence. *Rede Brasil AVC* recommends a minimum of 4 hours of team training per year. The World Stroke Organization and Rede Brasil AVC offer an online training platform for Stroke Centers ( *https://avc.encontrodigital.com.br/* ). This platform provides free online courses, certifications for the application of evaluation tools, live sessions, and activities on clinical treatment and rehabilitation of patients with stroke. This material can contribute to the development of a continuing education program within hospitals. **Supplementary Table 2** ( **Supplementary Material I** ; online only) highlights some of the courses and training available on the platform. We also present the website where international certification for the application of NIHSS and ERm can be obtained.

The expansion of online education is crucial to ensuring that physical therapists in remote areas or those with limited access to training centers can receive adequate education. However, online training may not be the most suitable method for developing practical skills. We recommend that, within the resources and possibilities available in each region, physiotherapists and hospital managers pursue hands-on training opportunities, particularly to address the practical skills needed for complex mobilization scenarios involving patients with varying degrees of impairment.

## GUIDELINES FOR HOSPITAL DISCHARGE

Individuals who have had a stroke often experience motor, sensory, and/or cognitive impairments that significantly affect their lifestyle and overall quality of life. Consequently, it is crucial for a multidisciplinary team in the stroke unit to provide comprehensive guidance on patient care beyond hospital stay. It is recommended that the guidance process for both patients and caregivers commence upon admission and continues throughout the hospitalization period until discharge, to avoid an overwhelming amount of information on the day of discharge, which could be detrimental. This process should involve a multi-disciplinary approach.

**Supplementary Figure II** ( **Supplementary Material I** ) outlines the key areas that the physiotherapy team should address during the guidance sessions with patients and their family members. These points are presented in the form of a checklist that should be completed before the patient is discharged from the hospital. This ensures that the essential aspects of care will be thoroughly discussed and understood by patients and caregivers. The use of the checklist facilitates the process of identifying individual needs and ensuring appropriate referrals by the multidisciplinary team.

We recommend that patients and caregivers not only receive guidelines during physiotherapy, but also actively participate in training sessions. These sessions should involve the provision of manuals or educational materials containing information about the disease, significance of hospitalization in a specialized stroke care unit, and guidance on postdischarge care. Healthcare professionals must explain the content of these materials and address any questions or concerns that may arise. For patients with limited mobility, it is particularly important to provide practical demonstrations and training sessions on proper positioning techniques and transfers. This hands-on approach will facilitate a better understanding and application of the training content, increasing caregivers' confidence and competence in performing essential tasks such as transfers and personal care.

Caregivers play an important role in patient care after stroke and are crucial to the successful transition from hospital to home. Their primary responsibilities include environmental adaptations, social support, assistance with mobility, and activities of daily living. 66 The caregiving burden is substantial, often resulting in significant mental health impacts.

To support this challenging role, we developed **Supplementary Table 3** ( **Supplementary Material I** ; (online only) to provide resources designed to guide families and caregivers, aiming to improve the quality of care after hospital discharge. Many of these materials were prepared in plain language to ensure accessibility across diverse social contexts. The postdischarge manuals include guidance on maintaining proper posture at home and preventing complications after a stroke. Additionally, we provide a link to the website and YouTube channel of Associação Brasil AVC, which features videos on positioning, transfer techniques, and mobility exercises for home practice. The table also includes a list of patient associations that offer free emotional support and guidance to patients and their families, further enhancing the support network available postdischarge.

The transition of care from hospital to home is a complex issue that requires further study in the Brazilian context. A systematic review published by Cochrane in 2021 suggests that providing information actively to patients improves stroke knowledge and reduces anxiety. 67 However, a recent meta-analysis published in 2024 analyzed the effectiveness of different interventions in reducing caregiver burden and found no significant effect. 68 Thus, there is still a lack of studies proving the best intervention and its timing. For this reason, we chose to suggest educational materials and adopt an active approach in which the professional identifies the patient's and caregiver's needs, provides guidance, and offers opportunities to clarify doubts and reinforce instructions.

It is highly recommended that rehabilitation services be accessed promptly after hospital discharge, as there is an optimal period for functional recovery characterized by heightened neuroplasticity. This critical phase occurs between the acute stage and the early subacute phase of stroke (7 days to 3 months). 6 Therefore, it is imperative that stroke units collaborate with local health authorities to prioritize posthospital discharge rehabilitation as an essential component of stroke patient care. By ensuring effective coordination, patients will have improved access to rehabilitation services, thereby maximizing their chances of optimal recovery and rehabilitation outcomes.

## QUALITY INDICATORS FOR PHYSIOTHERAPY AFTER A STROKE

Quality indicators are valuable tools in health management. In **Appendix 5** ( **Supplementary Material I** ; online only), we propose a list of indicators specifically tailored to physiotherapy services.

### Study Limitations

International studies show that proper care transition from hospital to home increases the chances of functional independence and recovery after stroke. 69 However, in Brazil, there is a need for further studies on effective models of care transition. Although we acknowledge the importance of this issue, the focus of the protocol was to optimize physiotherapy interventions during the hospital phase, without discussing in detail the transition of care to the home setting. We included a hospital discharge checklist to guide physiotherapists in identifying the patient's needs and assisting with appropriate referrals for rehabilitation after discharge. We provide links to websites with information for families on how to care for stroke patients at home. Additionally, we recommend that the hospital team conducts follow-ups 3 months poststroke to assess the number of patients who were able to continue rehabilitation. This allows the multidisciplinary team to develop local strategies to ensure continuity of care.

Another limitation of the current protocol is the need for future studies to evaluate its feasibility in different regions of the country. Given Brazil's vast geographical extent, stroke care is influenced by the social inequalities present in the country: while more prosperous regions can afford high-quality resources, underserved areas face severe limitations in access to stroke prevention, treatment, and rehabilitation. 70 Therefore, studying the implementation of the protocol across Brazil and addressing regional specificities is of utmost importance.

### Portuguese Version of the Protocol

In **Supplementary Material II** (online only; available at https://www.arquivosdeneuropsiquiatria.org/wp-content/uploads/2025/02/ANP-2024.0096-Supplementary-Material-2.pdf ), we present the Portuguese version of this protocol.
